# Beneficial effects of dantrolene in the treatment of rhabdomyolysis as a potential late complication associated with COVID-19: a case report

**DOI:** 10.1186/s40001-021-00489-8

**Published:** 2021-02-08

**Authors:** Nobutaka Chiba, Masakazu Matsuzaki, Takayuki Mawatari, Minori Mizuochi, Atsushi Sakurai, Kosaku Kinoshita

**Affiliations:** 1 Division of Emergency and Critical Care Medicine, Department of Acute Medicine, Nihon University School of Medicine, 30-1 Oyaguchi-kamimachi, Itabashi-Ku, Tokyo, 173-8610 Japan; 2 Department of Emergency and Critical Care Medicine, Nihon University Hospital, Tokyo, Japan; 3 Department of Internal Medicine, Kanamachi Kisen Hospital, Tokyo, Japan

**Keywords:** COVID-19, Rhabdomyolysis, Hyperpyrexia, Dantrolene, Immune response

## Abstract

**Background:**

Patients with severe COVID-19 have disorders of the respiratory, cardiovascular, coagulation, skeletal muscle, and central nervous systems. These systemic failures may be associated with cytokine release syndrome, characterized by hyperpyrexia, thrombocytopenia, hyperferritinemia, and the elevation of other inflammatory markers. Rhabdomyolysis with high fever is a complication that is rarely found in COVID-19. The exact relations of these clinical conditions in patients with COVID-19 remain unknown.

**Case presentation:**

We present the case of a 36-year-old man with severe COVID-19 complicated by rhabdomyolysis and high fever. After admission, his condition continued to deteriorate, with a high body temperature. On day 9, the patient had elevated creatine kinase and myoglobin levels consistent with rhabdomyolysis (26,046 U/L and 3668 ng/mL, respectively). In addition to viral therapy, he was immediately treated with hydration. However, the patient had persistent fever and elevated creatine kinase levels. The patient was diagnosed with malignant hyperthermia as a late complication of COVID-19, although he had no hereditary predisposition to malignant hyperthermia or neuroleptic malignant syndrome. The administration of dantrolene with muscle relaxation and anti-inflammatory function showed potential efficacy for rhabdomyolysis, high fever, and increased plasma inflammatory markers.

**Conclusions:**

Malignant hyperthermia is triggered by not only anesthetic agents but also viral infections. A possible mechanism of malignant hyperthermia is hypersensitivity of calcium release from the sarcoplasmic reticulum. These include mutations in or the activation of the skeletal muscle ryanodine receptor calcium release channel. Dantrolene is a ryanodine receptor antagonist and is used as an anti-inflammatory agent. The administration of dantrolene showed potential efficacy for rhabdomyolysis, high body temperature due to inflammation, and increased inflammatory markers. The underlying mechanism of the association of rhabdomyolysis and high fever in COVID-19 might be similar to the pathogenesis of malignant hyperthermia.

## Background

The majority of patients with severe coronavirus disease 2019 (COVID-19) have acute respiratory distress syndrome and lymphopenia; in addition, some have disorders of the central or peripheral nervous system, cardiac arrhythmias, cardiomyopathy, rhabdomyolysis, coagulopathy, and shock [1]. These systemic failures may be associated with cytokine release syndrome and characterized by hyperpyrexia, thrombocytopenia, hyperferritinemia, and the elevation of other inflammatory markers [2]. We present the case of a 36-year-old man with severe COVID-19 complicated by rhabdomyolysis and high fever who was administered dantrolene. As a result, not only reductions in creatine kinase (CK) and myoglobin levels and body temperature but also decrease in inflammatory markers, including C-reactive protein (CRP) and ferritin, were found.

## Case presentation

A 36-year-old previously healthy man presented to the emergency department with 2 days of worsening dyspnea. He had fever, cough, and fatigue during the week before presentation. His initial vital signs included temperature 39.2 °C, blood pressure 141/105 mmHg, respiratory rate 22 breaths per minute, heart rate 142 beats per minute, and oxygen saturation 92% on room air. Physical examination revealed mild crackles in both basal lung fields. He denied myalgia. Pertinent laboratory findings included the following: white blood cell count 5.7 × 10^3^/μL (reference, 3.3–8.6 × 10^3^/μL), lymphocyte count 11.7% (reference, 25–55%), platelet count 165 × 10^3^/μL (reference, 158–348 × 10^3^/μL), CRP 3.17 mg/dL (reference, 0.02 mg/dL or less), lactate dehydrogenase 220 U/L (reference, 124–222 U/L), CK 170 U/L (reference, 59–248 U/L), myoglobin 20 ng/mL (reference, 20–85 ng/mL), creatinine 0.7 mg/dL (reference, 0.65–1.07 mg/dL), D-dimer 0.7 ng/mL (reference, 1 ng/mL or less), and brain natriuretic peptide 3.9 pg/mL (reference, 18.4 pg/mL or less). Chest radiography showed patchy bilateral opacities in the lung parenchyma, and computed tomography of the chest showed bilateral ground-glass opacities. We treated the patient with oxygen inhalation, azithromycin, and ceftriaxone, and with antipyretics to control his high fever. On day 2 after admission, a reverse-transcriptase polymerase chain reaction assay detected the presence of severe acute respiratory syndrome coronavirus 2 (SARS-CoV-2) RNA by nasopharyngeal swab. The next day, his condition deteriorated, with hypoxemia. We started him on favipiravir [3600 mg on day 1 and 1600 mg per day on day 2 and subsequently, median treatment 14 days (IQR, 12 to 14 days)]. Anti-inflammatory agents, such as steroids and tocilizumab, were not administered during admission. On day 4 after admission, his condition continued to deteriorate, with fever, hypotension, and hypoxemia. He was administered midazolam, fentanyl, and rocuronium, and underwent endotracheal intubation. He was admitted to the intensive-care unit (ICU).

After ICU hospitalization, sedative therapy with dexmedetomidine was used for mechanical ventilation support. His condition continued to deteriorate, with high fever and high ventilatory requirements. High body temperature and CRP levels, systemic inflammatory response syndrome, and refractory hypoxemia (PaO_2_:FiO_2_ 159 mmHg and FiO_2_ of 0.6 with PEEP 15 cmH_2_O) continued.

**Fig. 1:**
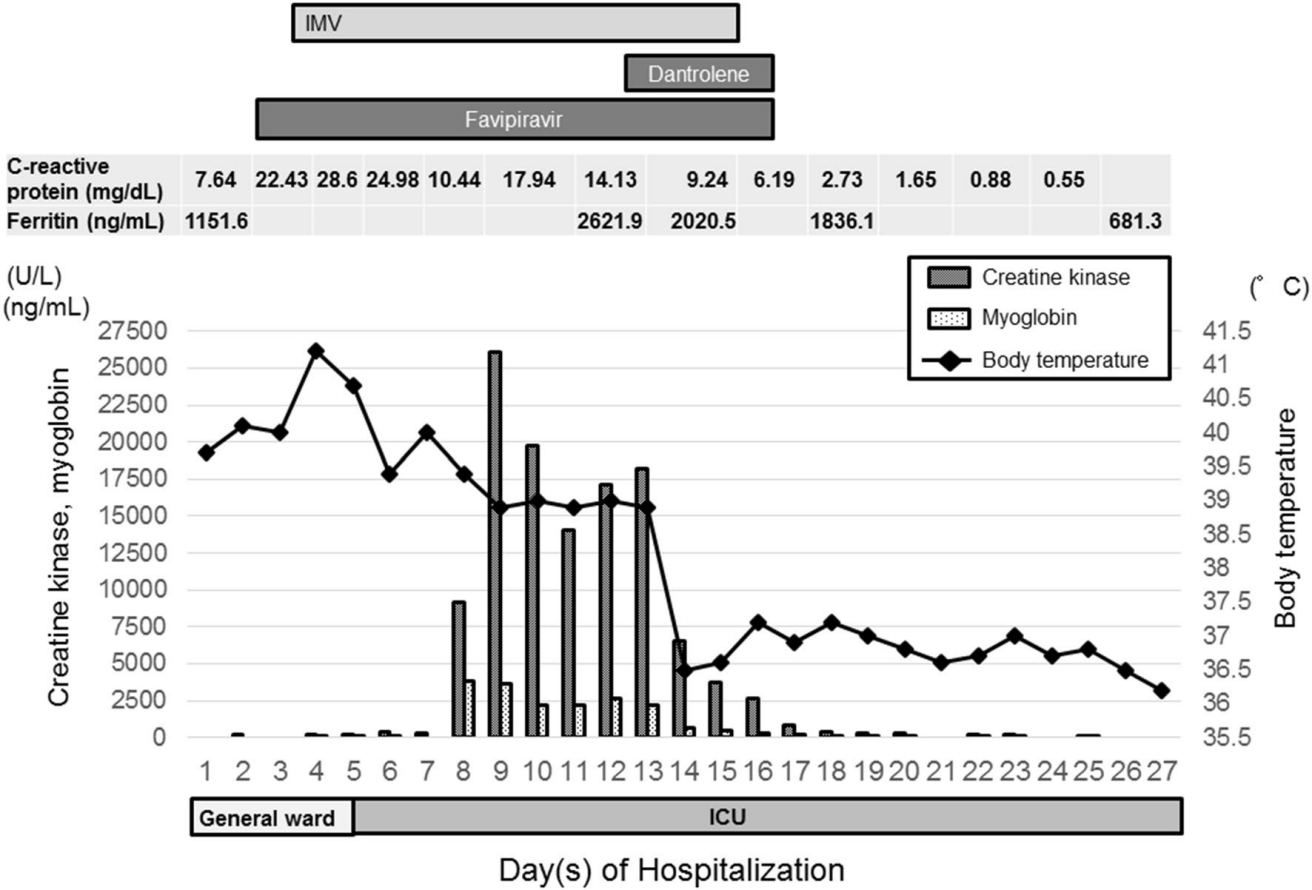
Clinical course of the patient. Effect of dantrolene administration on creatine kinase, myoglobin, and body temperature. Reductions in inflammatory markers, including C-reactive protein and ferritin, were found

On hospital day 9, his laboratory examination revealed that CK and myoglobin levels started to increase (to 26,046 U/L and 3668 ng/mL, respectively) (Fig. 1), and he developed muscle rigidity on the jaw and extremities in addition to tachycardia, tachypnea, and diaphoresis. Blood gas analysis showed that PaCO_2_ was 51 mm Hg (reference 35–45 mm Hg). His other laboratory examinations were normal: sodium, 144 mmoL/L (reference 138–145 mmoL/L); potassium, 4.7 mmoL/L (reference 3.6–4.8 mmoL/L); phosphorous, 3.2 mg/dL (reference 2.7–4.6 mg/dL); thyroid stimulation hormone (TSH), 0.94 (reference 0.34–3.8 μIU/mL); free triiodothyronine (FT_3_), 2.21 (reference 2–3.8 pg/mL); and free thyroxine (FT_4_), 1.04 (reference 0.8–1.5 ng/dL). Fatty-acid oxidation disorder was unlikely due to normal free carnitine [50.6 μmol/L (reference, 36–74 μmol/L)] and acyl carnitine [9.2 μmol/L (reference, 6–23 μmol/L)] levels. The patient was diagnosed with malignant hyperthermia associated with a late complication of COVID-19, although he had no hereditary predisposition to malignant hyperthermia or neuroleptic malignant syndrome. In addition to ongoing treatment, he received a total of a 3-L bolus of intravenous crystalloids. After fluid therapy, CK and myoglobin levels tended to decrease (to 14,047 U/L and 2179 ng/mL, respectively). However, on the next day, although he continued to receive fluid resuscitation, his CK and myoglobin levels were elevated again (to 18,205 U/L and 2612 ng/mL, respectively), and his high body temperature and CRP and ferritin levels persisted (Fig. 1). On the other hand, compartment syndrome was unlikely because his color of skin and pulse of the extremities revealed no abnormalities. We started him on 20 mg dantrolene intravenously four times a day from 14 to 16 days after admission and 50 mg orally three times a day over the next 2 days. Consequently, his body temperature started to decrease, and he showed a decrease in CK, myoglobin, CRP, and ferritin levels (Fig. 1). It was possible to extubate him on the 17th day after admission. His clinical condition continued to improve. After a negative SARS-CoV-2 test, we finally discharged him to his home on the 28th day after admission.

## Discussion and conclusions

There have been some case reports of high fever and rhabdomyolysis associated with COVID-19 [3, 4]; however, the exact relations of these clinical conditions in patients with COVID-19 remain unknown. In our case, we diagnosed malignant hyperthermia with rhabdomyolysis, and the patient was administered dantrolene. After the administration of dantrolene, his fever and serum CK and myoglobin levels returned to the normal range.

Malignant hyperthermia is triggered by various stimulations, such as anesthetic agents, heat stroke [5], and viral infections [6]. A possible mechanism of malignant hyperthermia is hypersensitivity of calcium release from the sarcoplasmic reticulum. These include mutations in [7] or the activation [8] of the skeletal muscle ryanodine receptor calcium release channel. In the presence of mutations in or the activation of the ryanodine receptor, skeletal muscle cells can release proinflammatory cytokines, such as IL-6, and exhibit excessive muscle contraction, which might lead to rhabdomyolysis with heat production, resulting in high fever and serum CK and myoglobin levels in the clinic, as noted in the previous reports [6–8].

Dantrolene is essential to achieve the best possible outcome for patients with malignant hyperthermia and neuroleptic malignant syndrome, and these effects of dantrolene have also been demonstrated to reduce CK levels and high body temperature immediately in the previous reports [6, 9]. Indeed, dantrolene is a ryanodine receptor antagonist and is used as an anti-inflammatory agent [10]. The effectiveness of dantrolene is attributed to its ability to act on a Ca-releasing channel from the sarcoplasmic reticulum in skeletal muscle fibers, inhibit Ca release, and block interleukin (IL)-6, which is known as an endogenous pyrogen [8]. The proinflammatory cytokines IL-1β, IL-6, and IL-18 are inflammatory markers that are responsible for high fever and elevated plasma levels of CRP and ferritin in the clinic [11]. In particular, IL-6 can also be used as a marker to predict SARS-CoV-2 disease deterioration [12]. Previous studies demonstrated that dantrolene could prevent the activation of the ryanodine receptor and block IL-6 release [8]. In our patient, a decrease in CK and myoglobin levels was observed after the use of dantrolene with prompt alleviation of high fever. Interestingly, decreased plasma levels of CRP and ferritin were found simultaneously. In a recent report, it was suggested that hyperpyrexia may be caused by a SARS-CoV-2-related exuberant immune response. Therefore, hyperpyrexia with aggravated and excess immune responses becomes a predictor of worse outcomes in COVID-19 patients [13]. Therefore, it is suggested that the anti-inflammatory effects of dantrolene might contribute to resolving rhabdomyolysis and high fever in patients with COVID-19.

Case reports of patients diagnosed with SARS-CoV-2 infection presenting with rhabdomyolysis are rare [3, 4]. There have been several possible hypotheses explaining the pathogenesis of viral-induced rhabdomyolysis: direct viral invasion can lead to rhabdomyolysis, a robust immune response to viruses’ results in cytokine storms and damages muscle tissues, and circulating viral toxins may directly destroy muscle cell membranes [4, 14]. In a previous case report, two case reports of patients with simple pneumonia [3, 4] showed that an increase in CK following hospitalization was thought to be caused by myositis due to direct viral invasion and/or muscle twitching with shivering. Our patient presented with high fever, hyperferritinemia, and elevated CRP plasma levels, and rhabdomyolysis was observed with higher plasma CK levels. These clinical data improved to their normal ranges after the administration of dantrolene. Taken together, rhabdomyolysis is the result of ryanodine receptor activation and is not caused by myositis and/or muscle twitching. In the current report, the infection and replication of SARS-CoV-2 play a role in the activation of the ryanodine receptor of host cells, and it is suggested that dantrolene might have a potential effect [15]. On the other hand, whether our patient has an inherited malignant hyperthermia susceptibility trait or a potential predisposition remains unclear, because we could not perform some tests, such as the caffeine test, or gene examination.

In summary, rhabdomyolysis with high fever is a complication that is rarely found in COVID-19. Our case illustrates the role of dantrolene in COVID-19-associated rhabdomyolysis. The underlying mechanisms might be similar to the pathogenesis of malignant hyperthermia.

## Data Availability

Written informed consent was obtained from the patient for publication of this case report and any accompanying images. A copy of the written consent is available for review by the Editor-in-Chief of this journal. Given that no human experimentation was performed, no approval by an ethics board was required.
